# Selection of reference genes from *Shiraia bambusicola* for RT-qPCR analysis under different culturing conditions

**DOI:** 10.1186/s13568-016-0314-9

**Published:** 2017-01-03

**Authors:** Chen Zhang, Tong Li, Cheng-Lin Hou, Xiao-Ye Shen

**Affiliations:** College of Life Science, Capital Normal University, Beijing, 100048 People’s Republic of China

**Keywords:** *Shiraia bambusicola*, Reference genes, qRT-PCR, Reliability

## Abstract

Stable reference genes are necessary to analyse quantitative real-time reverse transcription PCR (qRT-PCR) data and determine the reliability of the final results. For further studies of the valuable fungus *Shiraia bambusicola*, the identification of suitable reference genes has become increasingly urgent. In this study, three conventional reference genes and nine novel candidates were evaluated under different light conditions (all-dark, all-light and 12-h light/dark) and in different media (rice medium, PD medium, and Czapek–Dox medium). Three popular software programs (geNorm, NormFinder and BestKeeper) were used to analyse these genes, and the final ranking was determined using RefFinder. *SbLAlv9*, *SbJsn1*, *SbSAS1* and *SbVAC55* displayed the best stability among the genes, while *SbFYVE* and *SbPKI* showed the worst. These emerging genes exhibited significantly better properties than the three existing genes under almost all conditions. Furthermore, the most reliable reference genes were identified separately under different nutrient and light conditions, which would help accessible to make the most of the existing data. In summary, a group of novel housekeeping genes from *S. bambusicola* with more stable properties than before was explored, and these results could also provide a practical approach for other filamentous fungi.

## Introduction

*Shiraia bambusicola* is an important and valuable macrofungus in the medical and food industries. It is noteworthy for its hypocrellins, the main secondary metabolites of *S. bambusicola*, whose use has been proposed for disease treatments that involve anti-clinical strains and anti-inflammatory and anti-viral activity (Su et al. 2011; Jiang et al. 2011; Zhou et al. 2009). Currently, to break the bottleneck of product yield and improve the understanding of biosynthetic pathways, the analysis of functional genes is attracting increasing attention (Deng et al. 2016).

Quantitative real-time reverse transcription PCR (qRT-PCR) is generally regarded as a convenient and efficient tool to analyse gene expression, but the RNA quantification, the reverse transcription reaction efficiency and other uncontrolled factors may limit the accuracy and stability of the final results (Huang et al. 2014; Hao et al. 2014). Thus, it is necessary to apply reliable reference genes to normalize the data.

A large number of research papers have been published on reference genes under different stresses or from different organs in plants (Warzybok and Migocka 2013; Lin et al. 2014), and similar works in filamentous fungi are gradually being conducted (Zampieri et al. 2014; Zhou et al. 2011). It is striking that most of the applied reference genes for fungi were directly copied from the existing results in plants or animals, such as *actin*, *tubulin* and *18S rRNA* (Fang and Bidochka 2006; Yan and Liou 2006). However, the divergent regulatory mechanisms and environmental stresses suggested that these reliable reference genes might not be suitable for the analysis of fungal gene expression. For example, the classical reference genes elongation factor (*EF*-*1*) and beta-tubulin (*β*-*tubulin*), which are widely used in plants, were not appropriate in *Hemileia vastatrix* (Vieira et al. 2011); another reliable reference gene, glyceraldehyde-3-phosphate dehydrogenase (*GAPDH*), used in several qPCR studies for the normalization of data, was not stable in *Heterobasidion annosum* (Raffaello and Asiegbu 2013). Thus, it is necessary to systemically rescreen the candidates in novel species from the fungal kingdom.

According to the current literature, exploring putative reference genes from the transcriptomic data is a feasible and easy assay in fungal species (Llanos et al. 2015; Nadai et al. 2015; Steiger et al. 2010). Likewise, there is a series of popular Excel-based software programs, such as geNorm, NormFinder and BestKeeper, that have been successfully used in previous studies (Vandesompele et al. 2009; Andersen et al. 2004; Pfaffl et al. 2004).

Thus far, research on *S. bambusicola* has focused on strain mutagenesis and product fermentation (Song et al. 2015). However, since the genome and transcriptome were published (Yang et al. 2014; Zhao et al. 2016), gene function and molecular regulation have become research hotspots. To make qRT-PCR results more reliable and the application of reference genes more widespread, we comprehensively rescreened the candidate reference genes for *S. bambusicola* under different light conditions and in different media.

## Materials and Methods

### Fungal strain and culture conditions

Strain zzz816 of *Shiraia bambusicola* was deposited in the China General Microbiological Culture Collection Centre (CGMCC, No: 3135). Fungal isolates adhered to agars were cultured in potato dextrose agar (PDA) medium (potato 200 g/L, dextrose 20 g/L, agar 15 g/L) at 26 °C for 7 days, and then the fresh mycelia were inoculated onto different media (rice medium: rice 800 g/L, PD medium: potato 200 g/L, dextrose 20 g/L, and Czapek–Dox medium: NaNO_3_ 3 g/L, K_2_HPO_4_ 1 g/L, MgSO_4_⋅7H_2_O 0.5 g/L, KCl 0.5 g/L, FeSO_4_ 0.01 g/L, sucrose 30 g/L) under three different light conditions, namely, 24 h of continuous darkness (all-dark), 24 h of continuous lighting with a light intensity of 1500 lx (all-light), and 12: a 12 h light photoperiod with 1500 lx light intensity (12-h light/dark).

Finally, each sample was collected at different time points: 2, 4 and 6 days.

### RNA isolation and cDNA synthesis

The total RNA of each sample was extracted from frozen mycelia using an improved method in our laboratory (Song et al. 2015), and the total RNA was dissolved in RNase-free water.

The first strand of cDNA was synthesized by reverse transcribing 1 μg of RNA with *TransScript* ^*®*^ All-in-One First-Strand cDNA Synthesis SuperMix for qPCR (One-Step gDNA Removal; TransGen, China). The quantity and quality of the total RNA extracted was determined using an Eppendorf BioPhotometer plus (Eppendorf, Ger), and the cDNA samples were stored at −20 °C.

### Primer design and quantitative real-time PCR

Primers were designed using Primer3 software (http://primer3.ut.ee/), and the specificity of the product was verified using 1% agarose gel electrophoresis and melting curves. The efficiency of the validated primer pairs remained at approximately 100% (Table 1). The qRT-PCR reaction was performed in LightCycler^®^480 multiwell plates (Roche Applied Science, Indianapolis, IN, USA) with a LightCycler^®^480II/96 (Roche Applied Science, Indianapolis, IN, USA) real-time PCR system using LightCycler^®^480 SYBR Green I Master Mix (Roche Applied Science, Indianapolis, IN, USA). The reactions were performed according to the recommendations of the manufacturer: 95 °C for 5 min for initial denaturation, followed by 45 thermal cycles of 10 s at 95 °C, 10 s at 60 °C and 20 s at 72 °C. The melting curve was performed with slow heating from 65 to 97 °C with continuous measurement of fluorescence 5 acquisitions per 1 °C. All reactions were performed with three biological and two technical replicates with negative controls. The qRT-PCR data were directly analysed using the “second derivative maximum” function in the LightCycler^®^480 Software Version 1.5 (Roche Applied Science, Indianapolis, IN, USA).

**Table 1:** Description of candidate reference genes, and the details of primers and amplicons

Gene	Description	Amplicon length (bp)	Primer sequence (5′ → 3′)	Efficiency (%)	R^2^
*SbSAS1*	GTP-binding protein SAS1	141	F: 5′-GCGATTCAGCGAAGACTCCT-3′	98	0.9998
			R: 5′-ATGGTCCTGAAACGCTCCTG-3′		
*SbTRX*	4A/4B type thioredoxin-like protein	184	F: 5′-TCAAGGCCATGTACGAGCTG-3′	100	1
			R: 5′-ACTAGACCCCTGCCCTTCTT-3′		
*SbtS*	t-SNARE	136	F: 5′-CACACAGTCCAAGTTGCAGC-3′	97	0.9999
			R: 5′-ATCGGTAGGTGATTGCGCAT-3′		
*SbJsn1*	RNA binding protein-like protein Jsn1	175	F: 5′-TGCCCAGAAGATCATCGACG-3′	101	0.9999
			R: 5′-ACGGCCAAGCATAACCTCAA-3′		
*SbCHP*	Conserved hypothetical protein	143	F: 5′-TACGTCATTGGTGTCCGAGC-3′	102	0.9990
			R: 5′-TTGCCTCGACATGGTCTTCC-3′		
*SbLAlv9*	LAlv9 family protein	159	F: 5′-TCCCCTCCAACAGCTCGATA-3′	97	0.9998
			R: 5′-TGACGAAGCGATGCAGAAGT-3′		
*SbPKI*	Pkinase-domain-containing protein	166	F: 5′-TGCCGCCATACTTCCAACTT-3′	97	0.9999
			R: 5′-TTATTTCCCGGAGAGCGGTG-3′		
*SbFYVE*	FYVE-domain-containing protein	167	F: 5′-GTGCAGGAGGATGGTTTGGA-3′	97	0.9998
			R: 5′-ACGCCCACACATACGACAAT-3′		
*SbVAC55*	Vacuolar protein sorting 55	151	F: 5′-GGCTGTCTTTCGTTCTTGCG-3′	98	0.9998
			R: 5′-AAGTCATCCCGGTTAGCTGC-3′		
*UBI*	Ubiquitin-activating enzyme	131	F: 5′-ATCGCTGGTCTGAGAGGTCT-3′	96	0.9997
			R: 5′-GGGTGGAGGAAGAATTGCGA-3′		
*VAC*	Vacuolar ATPase subunit 1	157	F: 5′-CCGTCATTGTTGCCGAGAAC-3′	99	0.9968
			R: 5′-CACACCAGCAGTCTCTTCGT-3′		
*TFC*	Transcription factor TFIIIC	170	F: 5′-CAAGGCCGAACTTAGCGATC-3′	101	0.9958
			R: 5′-CCTCAGCATCACCGTCATTG-3′		

### Statistical analyses

To select suitable reference genes, three software packages were used to calculate the stability: NormFinder, geNorm, and BestKeeper. An additional web-based tool, RefFinder, (http://fulxie.0fees.us/) was applied to integrate and rank all candidate reference genes (Xie et al. 2012).

To calculate the PCR amplification efficiencies (E) and correlation coefficients (R^2^) of each primer pair, standard curves were prepared using a 10-fold serial diluting plasmid, into which the reference gene was cloned in PEASY-T3^®^ (TransGen, China).

The efficiency (E) was calculated according to the equation *E* = (10^(−1/slope)^−1) × 100% (Radonić et al. 2004).

## Results

### Strategy for selecting reference gene candidates

In this study, twelve candidate reference genes appeared in the stabilization assay, and nine of them were involved in this test for the first time. The novel ones were sought out directly from the public transcriptome data sets (Zhao et al. 2016), and the selection was based on the ranking of the expression levels of each gene, expressed as reads per kilobase per million (RKPM). For the whole data set, we evaluated the coefficient of variation of RKPM, and the ones with relatively smaller coefficients were considered the genes of interest, including GTP-binding protein SAS1 (*SbSAS1*), 4A/4B type thioredoxin-like protein (*SbTRX*), t-SNARE (*SbtS*), RNA binding protein-like protein Jsn1 (*SbJsn1*), conserved hypothetical protein (*SbCHP*), LAlv9 family protein (*SbLAlv9*), Pkinase-domain-containing protein (*SbPKI*), FYVE-domain-containing protein (*SbFYVE*) and vacuolar protein sorting 55 (*SbVAC55*). Other three genes, ubiquitin-activating enzyme (*UBI*), vacuolar ATPase subunit 1 (*VAC*) and transcription factor TFIIIC (*TFC*), were generally used to normalize the qRT-PCR data and appeared to be especially reliable as reference genes in the preliminary study for *Shiraia bambusicola* (Song et al. 2015).

Before the analysis of expression stability, the gene-specific amplification of these genes was confirmed by the single-peak melting curves of the qRT-PCR products, and the primers provided a good reaction efficiency ranging between 96 and 102% (Table 1).

**Fig. 1:**
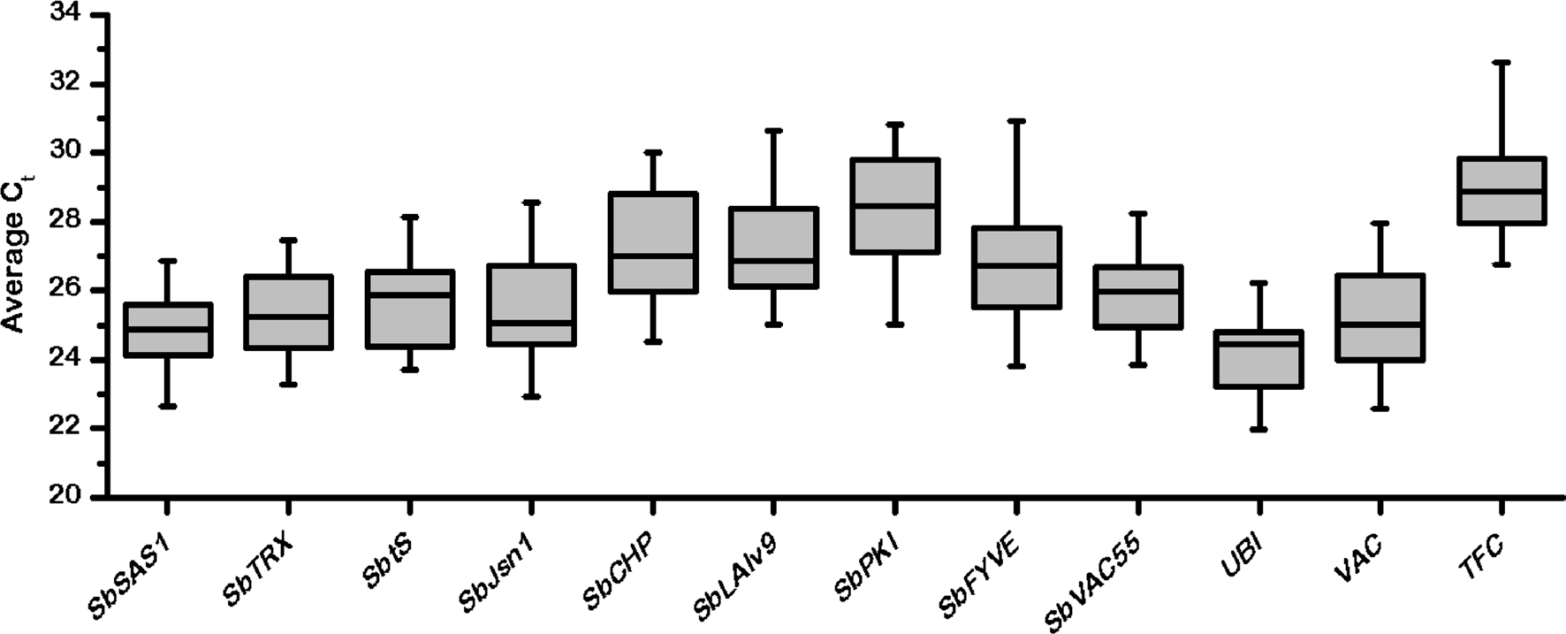
The range of CP values of 12 reference gene candidates for all samples. Each *box* indicates the 25th and 75th percentiles, and the caps represent the maximum and minimum values. The median is shown by the *line across the box*

The crossing point (CP) values were collected from all tested samples under different conditions and are shown in the box-plot (Fig. 1). The value of gene expression studied indicated a compact distribution and a limited range between 21 and 33. Among the genes, *TFC* displayed a lower expression variation, which mainly depended on the different media (Song et al. 2015).

### Expression stability analysis

Three different applets, geNorm, NormFinder, and BestKeeper, were applied to measure and rank the stability of candidate reference genes. The program geNorm (Steiger et al. 2010) classifies genes according to the control gene stability measure (M value), which represents the average of the pair-wise variation of a gene with all other control genes. NormFinder (Vandesompele et al. 2009) examines the stability of each single candidate gene independently and not in relation to the other genes. The results from geNorm and NormFinder can be compared easily because they both use raw data (relative quantities) as input data. BestKeeper (Andersen et al. 2004) is another Excel-based tool that determines the optimal reference genes using a pair-wise correlation analysis (Pearson correlation coefficient) of all pairs of candidate genes. It uses CP values (instead of relative quantities) as input and employs a different measure of expression stability from geNorm and NormFinder. However, this software is not able to analyse more than ten reference genes together, and thus the first ten ranking genes should depend on geNorm and NormFinder. The rankings of these software programs relied on different algorithms and were expected to lead to distinct outputs. Therefore, another software program, RefFinder (Radonić et al. 2004), was used to integrate the currently available major computational programs (geNorm, NormFinder, BestKeeper, and the comparative ΔΔCt method) to compare and rank the tested candidate reference genes. Based on the rankings from each program, it could assign an appropriate weight to an individual gene and calculate the geometric mean of their weights for the overall final ranking.

**Fig. 2:**
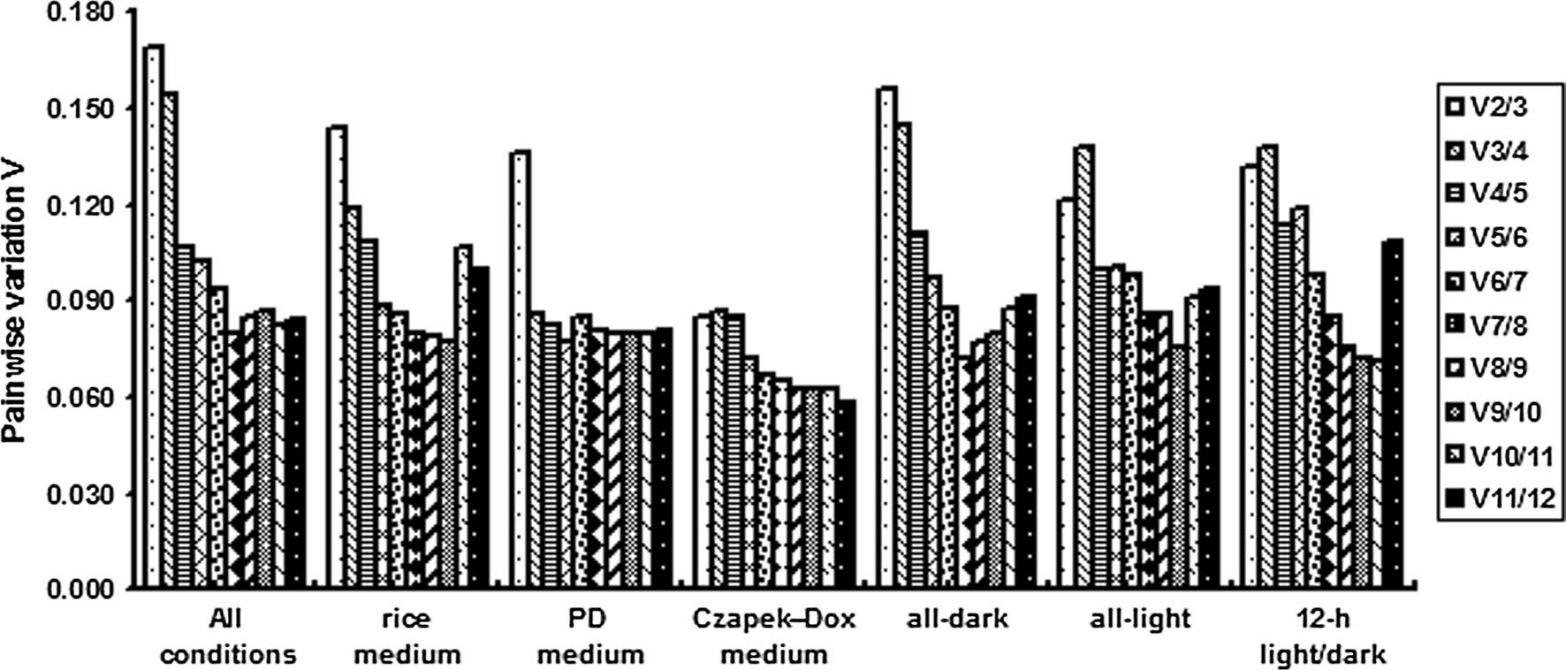
The geNorm-based results of the pairwise variation analysis. A sufficient number of genes n can be used for reliable normalization when Vn/n + 1<0.15

Using geNorm, *SbLAlv9*, *SbJsn1*, *SbCHP* and *SbSAS1* displayed the highest reliability overall (Table 2). In the rice medium with difference light conditions, *SbLAlv9*, *SbSAS1*, *SbJsn1* and *TFC* had a better effect than other candidate reference genes. In the PD medium, *SbLAlv9*, *SbJsn1*, *SbtS*, and *SbSAS1* ranked at the top positions, and in the Czapek-Dox medium, *SbJsn1*, *VAC*, *SbLAlv9* and *SbFYVE* were found to be the ideal reference genes. In the different light conditions, the best reference candidates were divided into three groups, including *SbLAlv9*, *SbJsn1*, *VAC* and *SbCHP* under all-dark conditions, *SbLAlv9*, *SbJsn1*, *SbCHP*, *SbtS* under all-light conditions and *SbLAlv9*, *SbJsn1*, *SbSAS1*, *SbtS* under the 12-h light/dark conditions. *SbFYVE* and *SbPKI* have been classified as the least reliable reference genes in most of the conditions. Furthermore, geNorm provided an output allowing a set of reliable normalization for the pairwise variation (V_n/n+1_) to help to determine the optimal number of reference genes (Fig. 2). Two reference genes were sufficient for most of the conditions, but the continuous darkness required a third reference gene. Four reference genes were suggested to ensure accurate all-round analysis of the nutrient and light conditions.

**Table 2:** Ranking of candidate reference genes calculated by geNorm according to different expression conditions

Ranking order^a^	All conditions	Different media			Different light conditions		
		Rice medium	PD medium	Czapek–Dox medium	All-dark	All-light	12-h light/dark
1	*SbLAlv9*	*SbLAlv9*	*SbLAlv9*	*SbJsn1*	*SbLAlv9*	*SbLAlv9*	*SbLAlv9*
	*SbJsn1*	*SbSAS1*	*SbJsn1*	*VAC*	*SbJsn1*	*SbJsn1*	*SbJsn1*
2	*SbCHP*	*SbJsn1*	*SbtS*	*SbLAlv9*	*VAC*	*SbCHP*	*SbSAS1*
3	*SbSAS1*	*TFC*	*SbSAS1*	*SbFYVE*	*SbCHP*	*SbtS*	*SbtS*
4	*SbtS*	*SbCHP*	*SbVAC55*	*SbCHP*	*SbTRX*	*SbSAS1*	*SbCHP*
5	*SbTRX*	*SbtS*	*SbTRX*	*SbSAS1*	*SbVAC55*	*SbFYVE*	*VAC*
6	*SbVAC55*	*SbVAC55*	*UBI*	*SbPKI*	*SbSAS1*	*SbTRX*	*UBI*
7	*VAC*	*SbTRX*	*SbCHP*	*UBI*	*SbtS*	*VAC*	*SbTRX*
8	*UBI*	*VAC*	*SbFYVE*	*TFC*	*SbPKI*	*SbVAC55*	*TFC*
9	*TFC*	*SbFYVE*	*VAC*	*SbTRX*	*TFC*	*UBI*	*SbVAC55*
10	*SbFYVE*	*UBI*	*TFC*	*SbVAC55*	*UBI*	*SbPKI*	*SbFYVE*
11	*SbPKI*	*SbPKI*	*SbPKI*	*SbtS*	*SbFYVE*	*TFC*	*SbPKI*

^a^Candidate reference genes were ranked from the most stable genes to the least stable genes

As shown in Table 3, *SbLAlv9*, *SbJsn1* and *SbSAS1* were identified as the best reference candidates by NormFinder, and *SbCHP*, *SbtS* and *VAC* displayed good properties for certain nutrient and light conditions. *SbFYVE* and *SbPKI* were ranked as the most unstable genes, and this result was also in agreement with the geNorm calculation.

**Table 3:** Ranking of candidate reference genes calculated by NormFinder according to different expression conditions

Ranking order^a^	All conditions	Different media			Different light conditions		
		Rice medium	PD medium	Czapek–Dox medium	All-dark	All-light	12-h light/dark
1	*SbLAlv9*	*SbLAlv9*	*SbJsn1*	*SbJsn1*	*SbVAC55*	*SbJsn1*	*SbLAlv9*
NF^b^	0.220	0.055	0.149	0.087	0.204	0.160	0.032
2	*SbJsn1*	*SbSAS1*	*SbSAS1*	*VAC*	*SbLAlv9*	*SbSAS1*	*SbSAS1*
NF	0.246	0.068	0.158	0.131	0.207	0.208	0.106
3	*SbSAS1*	*SbJsn1*	*SbtS*	*SbSAS1*	*VAC*	*SbtS*	*SbJsn1*
NF	0.252	0.259	0.172	0.212	0.255	0.249	0.169
4	*SbtS*	*SbVAC55*	*SbLAlv9*	*SbCHP*	*SbTRX*	*SbCHP*	*UBI*
NF	0.375	0.299	0.173	0.213	0.365	0.263	0.408
5	*SbCHP*	*TFC*	*SbVAC55*	*SbLAlv9*	*SbCHP*	*SbLAlv9*	*SbtS*
NF	0.376	0.356	0.215	0.235	0.367	0.311	0.410
6	*SbVAC55*	*SbCHP*	*SbTRX*	*UBI*	*SbJsn1*	*VAC*	*VAC*
NF	0.391	0.371	0.357	0.291	0.368	0.434	0.416
7	*VAC*	*VAC*	*UBI*	*SbPKI*	*SbSAS1*	*UBI*	*SbVAC55*
NF	0.401	0.388	0.372	0.327	0.375	0.463	0.436
8	*SbTRX*	*SbtS*	*SbCHP*	*SbFYVE*	*SbtS*	*SbVAC55*	*SbCHP*
NF	0.434	0.430	0.453	0.340	0.415	0.472	0.439
9	*UBI*	*SbTRX*	*SbFYVE*	*SbTRX*	*SbPKI*	*SbTRX*	*TFC*
NF	0.530	0.508	0.472	0.412	0.452	0.475	0.480
10	*TFC*	*SbFYVE*	*VAC*	*SbVAC55*	*TFC*	*SbFYVE*	*SbTRX*
NF	0.584	0.608	0.543	0.428	0.511	0.500	0.496
11	*SbFYVE*	*UBI*	*TFC*	*TFC*	*UBI*	*SbPKI*	*SbFYVE*
NF	0.621	0.759	0.637	0.444	0.699	0.726	0.577
12	*SbPKI*	*SbPKI*	*SbPKI*	*SbtS*	*SbFYVE*	*TFC*	*SbPKI*
NF	0.688	0.821	0.665	0.473	0.747	0.773	0.889

^a^Ranking of 12 candidate reference genes under different conditions from the most stable genes to the least stable genes by NF value

^b^The NF values were calculated by NormFinder, and the minimal NF value is considered to be the most stable

In contrast to geNorm and NormFinder, the BestKeeper algorithm is based on the coefficient of variance (CV) and the standard deviation (SD) calculated by the average CP value of each reaction. Moreover, we removed *SbFYVE* and *SbPKI* due to the higher variations based on geNorm and NormFinder. (Table 4). *SbVAC55*, *SbSAS1*, *UBI* and *SbTRX* were the four best reference genes among all the samples. Based on the different points, *SbSAS1*, *TFC*, *UBI* and *SbTRX* were the most stable on the different media, and *SbVAC55*, *UBI*, *SbSAS1* and *SbtS* were identified as suitable reference genes for the different light conditions, including all-dark, all-light and 12-h light/dark.

**Table 4:** Ranking of candidate reference genes were calculated by BestKeeper according to different expression conditions

Ranking order^a^	All conditions	Different media			Different light conditions		
		Rice medium	PD medium	Czapek–Dox medium	All-dark	All-light	12-h light/dark
1	*SbVAC55*	*SbSAS1*	*TFC*	*UBI*	*SbVAC55*	*SbVAC55*	*UBI*
CV^b^ ± SD^c^	3.3 ± 0.86	1.30 ± 0.32	2.39 ± 0.70	3.64 ± 0.89	2.11 ± 0.54	3.37 ± 0.88	3.44 ± 0.83
2	*SbSAS1*	*SbLAlv9*	*SbtS*	*TFC*	*SbSAS1*	*SbtS*	*SbTRX*
CV ± SD	3.60 ± 0.89	1.87 ± 0.49	2.97 ± 0.76	3.54 ± 1.07	2.40 ± 0.59	3.97 ± 1.02	3.39 ± 0.86
3	*UBI*	*SbVAC55*	*SbTRX*	*SbTRX*	*VAC*	*UBI*	*SbSAS1*
CV ± SD	3.88 ± 0.94	1.89 ± 0.49	3.19 ± 0.82	4.02 ± 1.09	2.86 ± 0.71	4.21 ± 1.02	4.12 ± 1.01
4	*SbTRX*	*TFC*	*SbSAS1*	*SbJsn1*	*SbtS*	*SbSAS1*	*TFC*
CV ± SD	3.83 ± 0.97	1.90 ± 0.53	3.43 ± 0.85	4.42 ± 1.16	2.83 ± 0.73	4.18 ± 1.04	3.72 ± 1.07
5	*SbtS*	*SbJsn1*	*SbVAC55*	*SbSAS1*	*SbLAlv9*	*TFC*	*SbVAC55*
CV ± SD	3.99 ± 1.02	2.21 ± 0.54	3.58 ± 0.93	4.86 ± 1.21	2.74 ± 0.75	4.20 ± 1.23	4.32 ± 1.12
6	*TFC*	*VAC*	*UBI*	*VAC*	*SbTRX*	*SbTRX*	*SbLAlv9*
CV ± SD	3.66 ± 1.06	2.29 ± 0.56	4.05 ± 0.99	4.75 ± 1.21	3.09 ± 0.78	5.02 ± 1.29	4.35 ± 1.17
7	*SbJsn1*	*SbTRX*	*SbLAlv9*	*SbVAC55*	*SbCHP*	*SbCHP*	*SbJsn1*
CV ± SD	4.53 ± 1.15	2.48 ± 0.61	3.66 ± 1.01	4.65 ± 1.22	2.94 ± 0.81	4.75 ± 1.30	4.67 ± 1.17
8	*SbLAlv9*	*SbtS*	*SbJsn1*	*SbLAlv9*	*SbJsn1*	*SbJsn1*	*VAC*
CV ± SD	4.28 ± 1.17	2.54 ± 0.63	3.93 ± 1.01	4.50 ± 1.25	3.59 ± 0.91	5.38 ± 1.39	4.89 ± 1.22
9	*VAC*	*SbCHP*	*SbCHP*	*SbCHP*	*UBI*	*VAC*	*SbtS*
CV ± SD	4.71 ± 1.19	2.74 ± 0.72	3.93 ± 1.08	4.88 ± 1.37	3.89 ± 0.94	5.81 ± 1.49	4.95 ± 1.26
10	*SbCHP*	*UBI*	*VAC*	*SbtS*	*TFC*	*SbLAlv9*	*SbCHP*
CV ± SD	4.54 ± 1.24	3.26 ± 0.77	5.82 ± 1.49	5.26 ± 1.37	3.25 ± 0.95	5.69 ± 1.57	5.79 ± 1.56

^a^Reference genes were listed from the most stable to the least stable based on the values of CV and SD

^b^Coefficient of variance expressed as a percentage on the CP level

^c^Standard deviation of the CP

To integrate the results obtained from different algorithms, RefFinder was used to perform the final ranking. As illustrated in Table 5, *SbLAlv9* was singled out as the top candidate in the global consideration of different nutritional and light conditions, followed by *SbJsn1*, *SbSAS1* and *SbVAC55*. Nevertheless, under specific conditions, the gene order would be altered, and the reliability of proper reference genes might be reduced. Thus, we propose two different methods—one medium with different light conditions and one light condition with different media—to improve the utilization of reference genes in practical experiments.

**Table 5:** Ranking of the candidate reference genes calculated using RefFinder under different conditions

Ranking order^a^	All conditions		Different media						Different light conditions					
			Rice medium		PD medium		Czapek–Dox medium		All-dark		All-light		12-h light/dark	
	Gene	R^b^	Gene	R	Gene	R	Gene	R	Gene	R	Gene	R	Gene	R
1	*SbLAlv9*	1.68	*SbLAlv9*	1.32	*SbJsn1*	1.68	*SbJsn1*	1.41	*SbVAC55*	1.86	*SbJsn1*	1.68	*SbLAlv9*	1.57
2	*SbJsn1*	2.3	*SbSAS1*	1.41	*SbtS*	2.71	*VAC*	2.21	*SbLAlv9*	1.86	*SbtS*	2.91	*SbSAS1*	2.45
3	*SbSAS1*	2.91	*SbJsn1*	3.41	*SbSAS1*	2.83	*SbSAS1*	4.05	*VAC*	3.22	*SbSAS1*	2.99	*SbJsn1*	2.82
4	*SbVAC55*	3.98	*SbVAC55*	3.87	*SbLAlv9*	3.25	*UBI*	4.12	*SbJsn1*	3.94	*SbLAlv9*	4.16	*UBI*	3.44
5	*SbtS*	4.47	*TFC*	4.47	*SbVAC55*	5	*SbLAlv9*	4.53	*SbSAS1*	5.12	*SbCHP*	4.28	*SbtS*	5.18
6	*SbCHP*	5.36	*SbCHP*	6.34	*SbTRX*	5.05	*SbCHP*	5.76	*SbCHP*	5.32	*SbVAC55*	4.9	*SbTRX*	6.32
7	*SbTRX*	6.26	*VAC*	7.17	*TFC*	6.04	*SbTRX*	6.34	*SbTRX*	5.38	*UBI*	6.19	*VAC*	6.45
8	*UBI*	6.84	*SbtS*	7.44	*UBI*	6.74	*TFC*	7.38	*SbPKI*	6.84	*VAC*	7.5	*SbVAC55*	7.27
9	*VAC*	7.91	*SbTRX*	8.21	*SbCHP*	8.46	*SbFYVE*	7.44	*SbtS*	7.11	*SbTRX*	7.64	*TFC*	7.35
10	*TFC*	8.8	*SbFYVE*	10.47	*SbFYVE*	9	*SbPKI*	7.65	*TFC*	10.47	*SbFYVE*	8.57	*SbCHP*	7.61
11	*SbPKI*	11.17	*UBI*	10.74	*VAC*	10.47	*SbVAC55*	10.36	*UBI*	11	*TFC*	9.64	*SbFYVE*	10.74
12	*SbFYVE*	11.24	*SbPKI*	11.74	*SbPKI*	11.74	*SbtS*	10.69	*SbFYVE*	11.17	*SbPKI*	10.74	*SbPKI*	11.74

^a^Genes were ranked according to their R values

^b^The R values were calculated by RefFinder to integrate the results from different programs, and a gene with more stable expression is expressed as a smaller number

### Application in detailed conditions

Although the appropriate reference genes had been presented as in Table 5, it was still inconvenient to search for a target effectively for one particular condition. To optimize the application of the ideal reference genes, we reprocessed the data and characterized the reference genes as a two-way table (Table 6). Therein, we could find the suitable reference genes more easily and accurately. For example, under continuous darkness (Table 5) and limited to rice medium, *SbVAC55* or *SbLAlv9* was sufficient for qRT-PCR analysis (Table 6).

**Table 6:** The most stable reference genes under different nutritional and light conditions

	Rice medium	PD medium	Czapek–Dox medium
All-dark	*SbVAC55* *SbLAlv9*	*SbtS* *SbVAC55*	*SbSAS1* *SbLAlv9*
All-light	*SbJsn1* *SbLAlv9*	*SbTRX* *SbSAS1*	*SbJsn1* *VAC*
12-h light/dark	*VAC* *SbSAS1*	*UBI* *SbSAS1*	*SbJsn1* *VAC*

## Discussion

Of all the candidate reference genes, three familiar genes (*UBI*, *VAC* and *TFC*) had been approved previously (Song et al. 2015), and another nine (*SbSAS1*, *SbTRX*, *SbtS*, *SbJsn1*, *SbCHP*, *SbLAlv9*, *SbPKI*, *SbFYVE*, *SbVAC55*) first emerged for the reliability of gene expression. Three new genes, *SbLAlv9*, *SbJsn1* and *SbSAS1*, indicated appropriate properties for all the nutrient and light conditions. *SbtS* and *SbVAC55* also separately showed high reliability for certain conditions. In contrast, only one old reference gene, *VAC,* displayed good activity for Czapek–Dox medium or all-dark conditions. As illustrated by these results, the exploration of new reference genes could indeed provide the more reliable genes and improve the assurance of the stability of gene expression in *S. bambusicola*. Furthermore, the discovery of novel reference genes might contribute to the use of reference genes in other species.

We chose typical nutrient and light conditions in this study that generally cover the culturing conditions of *S. bambusicola* or other species. Therefore, the reliable reference genes in this work were anticipated to be broadly used in the gene expression analysis of *S. bambusicola* and could also provide benefits for other fungal species. Furthermore, a table (Table 6) was constructed to facilitate the application of these results, with a two-way form to facilitate finding the stable reference genes for specific conditions. In this work, we tried our best to expand the scope of application and make the process more convenient.

In conclusion, nine novel candidate reference genes were introduced for the first time, and several of them (*SbLAlv9*, *SbJsn1*, *SbSAS1* and *SbVAC55*) were validated as ideal ones. In addition, this work provided a set of reference genes under different nutrient and light conditions, and these housekeeping genes are expected to be available in a more extensive range.

## Abbreviations

qRT-PCR: quantitative real-time reverse transcription PCR
EF-1: elongation factor
β-tubulin: beta-tubulin
GAPDH: glyceraldehyde-3-phosphate dehydrogenase
RKPM: reads per kilobase per million
SbSAS1: GTP-binding protein SAS1
SbTRX: 4A/4B type thioredoxin-like protein
SbtS: t-SNARE
SbJsn1: RNA binding protein-like protein Jsn1
SbCHP: conserved hypothetical protein
SbLAlv9: LAlv9 family protein
SbPKI: Pkinase-domain-containing protein
SbFYVE: FYVE-domain-containing protein
SbVAC55: vacuolar protein sorting 55
UBI: ubiquitin-activating enzyme
VAC: vacuolar ATPase subunit 1
TFC: transcription factor TFIIIC
CP: crossing point
CV: coefficient of variance
SD: standard deviation
